# The Impact of Weather on Influenza and Pneumonia Mortality in New York City, 1975–2002: A Retrospective Study

**DOI:** 10.1371/journal.pone.0034091

**Published:** 2012-03-28

**Authors:** Robert E. Davis, Colleen E. Rossier, Kyle B. Enfield

**Affiliations:** 1 Department of Environmental Sciences, University of Virginia, Charlottesville, Virginia, United States of America; 2 Division of Pulmonary and Critical Care, Department of Medicine, University of Virginia Health System, Charlottesville, Virginia, United States of America; National Institutes of Health, United States of America

## Abstract

The substantial winter influenza peak in temperate climates has lead to the hypothesis that cold and/or dry air is a causal factor in influenza variability. We examined the relationship between cold and/or dry air and daily influenza and pneumonia mortality in the cold season in the New York metropolitan area from 1975–2002. We conducted a retrospective study relating daily pneumonia and influenza mortality for New York City and surroundings from 1975–2002 to daily air temperature, dew point temperature (a measure of atmospheric humidity), and daily air mass type. We identified high mortality days and periods and employed temporal smoothers and lags to account for the latency period and the time between infection and death. Unpaired *t*-tests were used to compare high mortality events to non-events and nonparametric bootstrapped regression analysis was used to examine the characteristics of longer mortality episodes. We found a statistically significant (p = 0.003) association between periods of low dew point temperature and above normal pneumonia and influenza mortality 17 days later. The duration (r = −0.61) and severity (r = −0.56) of high mortality episodes was inversely correlated with morning dew point temperature prior to and during the episodes. Weeks in which moist polar air masses were common (air masses characterized by low dew point temperatures) were likewise followed by above normal mortality 17 days later (p = 0.019). This research supports the contention that cold, dry air may be related to influenza mortality and suggests that warning systems could provide enough lead time to be effective in mitigating the effects.

## Introduction

It is well known that intra-annual mortality exhibits a pronounced winter peak in locations with seasonal climates [1], [2]. In the United States, mortality arising from respiratory disease is 50% higher in winter than in summer [1]. One contributor to this excess winter mortality is influenza [3], the timing and severity of which can vary significantly from year to year. The association between the annual influenza peak and winter weather in temperate locations has lead to the hypothesis that weather variability could influence influenza mortality variations [4], [5].

Recent research examining climatic influences on influenza transmission has galvanized interest in this topic. Airborne transmission of Influenza A/Panama virus between guinea pigs was more likely at low temperatures and relative humidities [4]. Because temperature is physically/mathematically linked to relative humidity and thus confounds interpretation, a mass-based humidity measure (such as specific humidity or vapor pressure) provides a stronger relationship to influenza transmission [5]. Given the cold-season decline in specific humidity in temperate climates, a retrospective study [6] showed an association between year-to-year humidity variations and the timing of the influenza seasonal onset in U.S. states. This differs from tropical locations which generally lack a seasonal influenza peak [7]. One theory is that tropical climates are dominated by direct contact transmission which, unlike airborne transmission, is not influenced by air temperature and humidity [7].

These recent studies [4], [5], [6], [7] have motivated several reviews on climate and influenza seasonality [8]. [9], [10]. These reviews, which approach the issue from atmospheric sciences, virological, and epidemiological perspectives, do not reach firm conclusions on the causes of influenza seasonality but suggest that those causes are complex and multifactorial and that the solution will require interdisciplinary cooperation.

A variety of theories exist as to how weather and climate might exert some influence on influenza seasonality. Low temperatures enhance viral stability [4], reduce mucosal blood flow [11], and/or diminish mucociliary clearance [12]. Correlations exist between the number of upper respiratory infections and cold-air outbreaks [13]. It is commonly assumed that winter indoor crowding enhances influenza virus transmission, though direct evidence is lacking [8]. Low humidity conditions, which are often but not always accompanied by low temperatures, enhance survival times of viral aerosols [14], [15], [16]. If correct, these relationships suggest that indoor winter heating without humidification could enhance influenza transmission [8], as indoor absolute humidity tends to be correlated with outdoor values and thus is typically lower in winter [6].

From a micro-physical perspective, there is evidence that both droplet size and transmission mode depend on ambient environmental factors [17], [18]. Small droplets can remain airborne longer whereas large drops tend to precipitate, suggesting that ambient conditions would influence whether airborne- or contact-mode transmission predominates. The virulence of the influenza virus changes constantly as the virus undergoes antigenic shift and antigenic drift [8]. Although virulence depends upon a variety of factors, one possible atmospheric influence is that cold, dry air allows the lipid envelope encasing the influenza virus to remain intact longer, increasing the likelihood of infection [19].

Theories proposing that factors other than weather/climate are responsible for influenza seasonality include cycles in viral interference [20] and intrinsic temporal viral dynamics that operate independent of external forcing factors [21]. Some research suggests that the seasonality is driven by the school calendar in which students reconvene after a summer break, but this timing is not consistent with the typical onset of influenza in early winter [22].

We examine the hypothesis that cold and/or dry weather enhances human pneumonia and influenza (P&I) mortality through a retrospective study of daily mortality in New York City and environs from 1975–2002. We hypothesize that periods with colder and/or less humid conditions exhibit excess P&I mortality for a period of time following those climatic conditions. Our research differs from recent work on this topic that examined the timing of the influenza season onset over large geographic areas [6]—our focus is on the influence of daily weather on influenza characteristics for a single, large metropolitan area.

We selected New York City for this study for several reasons. Our study examines daily mortality, and statistical robustness is enhanced when the daily sample size is sufficiently large. Weather obviously has a high spatial variability, so it is important that the observed weather be representative of the environmental conditions likely experienced by the decedents. In addition, New York City's mid-latitude location provides a high degree of both interannual and intra-annual variability in weather and climate, so this variability provides a wider range of sample conditions. Thus, New York City has both a large enough population to provide a consistent daily mortality signal while the population density is high enough that the weather observed at a single station is sufficiently representative of conditions experienced throughout the metropolitan area.

## Methods

### Outcome Data

We conducted a retrospective cohort study of pneumonia and influenza (P&I) mortality of residents of the New York City metropolitan area. Daily frequencies of P&I mortality were tallied from National Center for Health Statistics archives for the New York City Consolidated Metropolitan Statistical Area (which, as defined in the year 2000, includes 30 counties in New York, New Jersey, Connecticut, and Pennsylvania). This period of record spans three revisions of the International Classification of Diseases (ICD) codes (Table 1).

**Table 1:** International Classification of Diseases periods and codes for pneumonia and influenza.

REVISION	DATES APPLICABLE	CODE
8th	1975–1979	470–474, 480–484
9th	1980–1998	480–484, 487
10th	1999–present	J10–J16, J18

In the National Center for Health Statistics mortality files that we used for this research, all information that could allow an individual to be identified has been removed. This research utilized only mortality counts for a large metropolitan area. These de-identified counts are stored in governmental archives for the purposes of retrospective research; because all personal identifying information is redacted, consent is not required. Thus, this research is exempt from IRB review under the auspices of Title 45 Part 46 exemption category 4.

Pneumonia or influenza must be listed as the primary cause of death to be included in this analysis. These diseases are commonly combined as an endpoint because of specific challenges associated with influenza. First, the number of deaths attributable to influenza is difficult to estimate directly because of a lack of virologically-confirmed infections. Second, many influenza-associated deaths occur from secondary complications when influenza viruses are no longer detectable by laboratory means [6], [23], [24], [25]. Third, the use of P&I mortality in the study of influenza also reflects the problems associated with other measures of prevalence of influenza. Reporting of cases of influenza through routine channels is unsatisfactory because mild influenza may be under-diagnosed and the use of laboratory confirmation is skewed by the impact of variable testing patterns based on prevalence of disease [26]. Finally, multiple studies have shown that there exists a relationship between influenza morbidity and P&I mortality that can be mathematically described and used in epidemiological studies [3], [27], [28], [29], [30], [31].

Daily deaths were aggregated into ten age groups (0–4, 5–14, 15–24, 25–34, 35–44, 45–54, 55–64, 65–74, 75–84 and >84) and standardized via direct standardization [32] based upon the age distribution of population of the United States in the year 2000. This procedure adjusts for temporal changes in age demographics over the period of record based on data for each county using U.S. Census archives, thereby allowing for consistent comparisons of mortality rates over time. After age standardization, the long-term mean for each day (within ICD period) was subtracted from that day's standardized P&I mortality to remove the inherent seasonal signal from the time series. Because our goal was to determine if cold and/or dry days or periods within a year were related to P&I mortality peaks, this level of granularity requires the examination of daily data with the seasonal signal removed.

**Figure 1:**
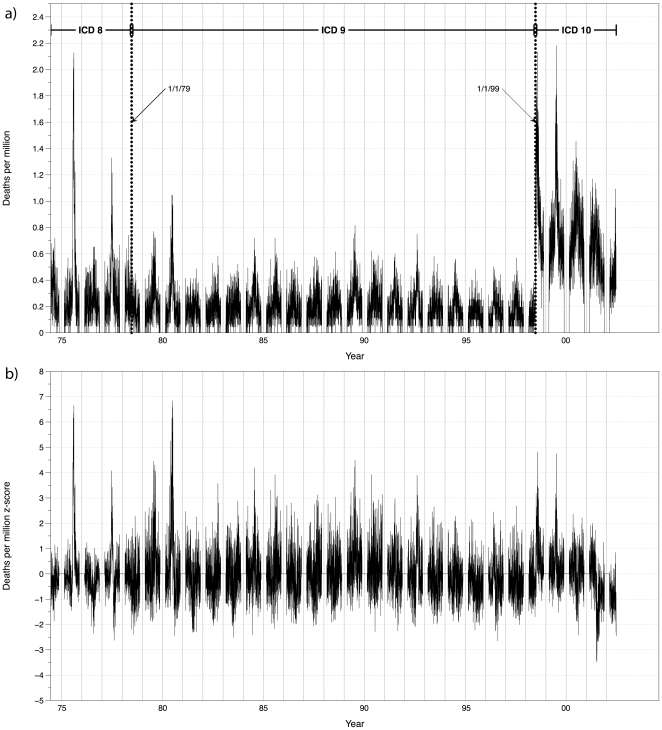
Time series of pneumonia and influenza mortality for New York City from September, 1975–May, 2002. a) (top) Daily age-standardized pneumonia and influenza mortality time series (deaths per million; June, July and August have been deleted). The relevant periods for the International Classification of Diseases (ICD) are identified by a thick vertical line; b) (bottom) Resulting mortality time series after removing the seasonality and converting to z-scores for each ICD period. Vertical dividers identify influenza seasons (September–May) with the year assigned to the January–May period (i.e., December, 1979 is in the 1980 flu “season,” labeled as “80” on the x-axis).

Examination of the P&I mortality time series exhibits obvious temporal discontinuities that are exactly coincident with the dates of ICD revision code changes. We removed this artifact by converting each day's P&I mortality to a z-score by dividing the mean departure by the standard deviation separately for each of the three relevant ICD periods (Table 1). The time series of age-standardized mortality prior to and after deseasoning and z-score adjustment is shown in Figure 1a and Figure 1b, respectively. Because of the general lack of influenza in the summer months, June, July, and August were removed from the analysis as they had the potential to distort any relationships during the primary influenza season.

Mortality data were smoothed using a 17-day leading moving average (e.g., mortality on January 1 is the mean from January 1–17). This smoother was selected after testing a variety of filter lengths and based upon prior research [6].

**Figure 2:**
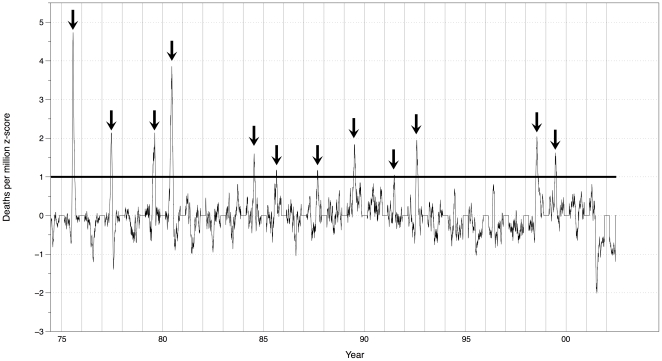
Identification of 12 mortality “episodes” that exceeded the z≥1 criterion. The time series is Figure 1b smoothed with a 17-day centered moving average filter. A centered smoother is used here to more clearly present the peak times of the mortality episodes (see Table 2).

We examined both daily P&I mortality “events” and longer mortality “episodes.” “Events” are days with (smoothed) mortality at least one standard deviation above the long-term (smoothed) mean for that date. This z≥1 criterion was chosen because the frequency distribution of smoothed mortality is positively skewed with the tail beginning at approximately one standard deviation. After smoothing, there is an obvious tendency for high mortality events to cluster into prolonged periods when the z≥1 threshold is exceeded (Figure 2). We thus identified 12 P&I mortality episodes over the 28-year period of record (Table 2).

**Table 2:** Characteristics of the 12 high mortality “episodes.”

SEASON	START	END	DURATION(DAYS)	TOTALDEATHS	AVERAGE DEATHS/DAY
1976	19-Jan	28-Mar	70	144.58	2.07
1978	14-Dec	2-Feb	51	66.84	1.31
1980	16-Jan	21-Mar	65	82.67	1.27
1981	22-Nov	6-Feb	77	137.21	1.78
1985	15-Jan	25-Feb	42	44.54	1.06
1986	27-Feb	26-Mar	28	28.60	1.02
1988	12-Mar	11-Apr	31	28.55	0.92
1990	27-Dec	19-Feb	55	66.19	1.20
1992	31-Dec	21-Jan	22	22.61	1.03
1993	30-Jan	23-Mar	53	65.68	1.24
1999	5-Jan	4-Mar	59	78.26	1.33
2000	16-Dec	1-Feb	47	51.29	1.09

Deaths are age-standardized deaths per million in z-score units. Total deaths and average deaths per day include the entire time period between the start and end of the episode.

To summarize the outcome data treatment, after age standardization the daily mortality data were deseasoned to remove the large influence of season on respiratory infection and converted to z-scores to adjust for discontinuities related to ICD coding. These data were then smoothed using a 17-day leading smoother to account for the inherent lag between infection and mortality. Days or periods with z-scores>1 were identified as mortality “events” or “episodes,” respectively (Figure S1).

### Weather Data

Hourly climate data from La Guardia Airport, New York, were retrieved from National Climatic Data Center archives. We utilize 1200 and 1900 Universal Time Coordinate (UTC) air temperature (T) and dew point temperature (T_d_) to approximate the typical times of the warmest and coldest hours of the day (7 or 8 a.m. and 2 or 3 p.m. local time). The dew point temperature is the temperature at which water vapor begins to condense via cooling at constant pressure. We use dew point as a measure of the amount of moisture in the air because, unlike relative humidity, it is independent of air temperature [5].

In addition to dew point temperature, we employ an air mass classification, which has the advantage of incorporating a variety of weather variables into a single nominal variable. Specifically, we utilize the Spatial Synoptic Climatology (SSC) [33] which classifies each day's weather into one of six air mass types [dry moderate (DM), dry polar (DP), dry tropical (DT), moist moderate (MM), moist polar (MP), moist tropical (MT)]. A seventh transition (TR) category identifies days characterized by a significant change in weather—typically a frontal passage [34]. The SSC uses air temperature, dew point temperature, wind, surface air pressure, and total cloud cover observations taken four times per day as input to the classification. Thus, the SSC is a multivariate nominal classification of daily weather conditions. The approach has been utilized in a variety of human health applications [35], [36], [37], [38], [39], [40], [41].

The temperature and dew point time series were converted to z-scores to remove seasonality and then smoothed using a centered 5-day moving average filter. This filter length was employed after examining various options because it represents a balance between high frequency weather events and more long-term (monthly to seasonal) trends.

The seasonality of the SSC air mass types was removed by comparing the presence (coded 1) or absence (coded 0) of each air mass type on each day of the year to the long-term average frequency. For example, if Moist Moderate air was present on average 30% of the time on January 1 over the period of record, then its occurrence on January 1, 2000 would result in a value of +0.7 for that day. These daily anomalies were then converted into continuous variables using a centered 7-day moving average filter for each SSC category.

In summary, raw dew point observations were first de-seasoned by conversion to z-scores and then smoothed using a 5-day filter. Daily air mass frequencies were converted from a nominal to a continuous variable by first adjusting for the long-term frequency on each day and then smoothing those frequencies using a 7-day moving average (Figure S2).

### Statistics

#### Daily Analyses

A series of *t*-tests were employed to determine if temperature, dew point, and air mass frequency differed between high P&I mortality events (z≥1) and non-events. To address the temporal autocorrelation in the weather variable time series and the resulting overestimate of the true degrees of freedom, the sample size was adjusted based upon the lag one temporal autocorrelation [42] (Wilks 2006) as follows:
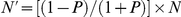
where N = number of observations

 N′ = adjusted degrees of freedom

 P = lag one temporal autocorrelation.

N′ was then adjusted again based on the length of the smoother employed to determine the final effective sample size. For these and all other tests, a Type I error rate of 0.05 was employed and Levene's test for equality of variances was used to determine if pooling of the samples was required.

The following tests were performed:

1. smoothed temperature, dew point, and air mass frequency, lagged 17-days, between mortality events (z≥1) and non-events (z<1) using an unpaired two-sample t-test (Figure S3);
2. same as in 1 for unsmoothed temperature and dew point temperature (to determine if a strict 17-day lag exists); and
3. same as in 1 but using a one-sample t-test (to account for the possible influence of disparate sample sizes between groups).

#### Episodic Analyses

For each high P&I mortality episode, we calculated the duration (in days), the summed total mortality over the entire episode, and the average daily episode mortality (Table 2). These quantities served as dependent variables in a linear regression analysis *vs.* the independent (weather) variables (1200 and 1900 UTC T and T_d_ and the deseasoned SSC frequencies). We used a 17-day lag in which the mean for each variable was calculated across the days in the episode and the preceding 17 days. This lag was selected based upon prior research that examined absolute humidity [6] and the results of other studies [28], [43].

Given the relatively small number of episodes, we used bootstrapped regression analysis to generate a robust estimate of the regression coefficients. Based on the initial full sample, data sets of the same size were generated by randomly sampling variable pairs, with replacement, and estimating the regression parameters from that sample using ordinary least-squares. This procedure was repeated 10,000 times and the resulting suite of regression coefficients was examined to determine if the 2.5 percentile and 97.5 percentile observations were of the same sign. If so, the regression slope was deemed to be statistically significant [44].

## Results

### Daily Analyses

In the daily analysis, 1200 UTC dew point temperature was significantly lower for events than for non-events (p = 0.003), a result that is consistent with the hypothesis that drier conditions are related to enhanced P&I mortality. However, 1900 UTC dew point was higher during events (p = 0.036), a result that contradicts the underlying hypothesis.

When this test was repeated without smoothing the weather variables, only 1200 UTC dew point was significant (p = 0.028; Table 3). The lack of a relationship for 1900 UTC dew point (p = 0.181) suggests that the finding of high afternoon dew points during mortality events using smoothed weather data was not robust.

**Table 3:** Results of *t*-tests comparing weather variables between mortality event days to non-event days.

	2-sample Smoothed			2-sample Unsmoothed			1-sample
	Event	Non-Event	p	Event	Non-Event	p	p
1200 UTC T	−0.114	−0.025	0.068	−0.120	−0.026	0.150	**0.018**
1200 UTC T_d_	**−0.372**	**0.003**	**0.003**	**−0.383**	**−0.248**	**0.028**	**0.000**
1900 UTC T	−0.012	−0.028	0.725	−0.012	−0.029	0.767	0.783
1900 UTC T_d_	**−0.053**	**−0.138**	**0.036**	0.056	−0.032	0.139	0.181
Dry Moderate	0.013	0.005	0.984	n/a	n/a	n/a	0.239
Dry Polar	−0.000	−0.008	0.277	n/a	n/a	n/a	0.994
Dry Tropical	0.001	0.002	0.998	n/a	n/a	n/a	0.561
Moist Moderate	**−0.026**	**0.001**	**0.000**	n/a	n/a	n/a	**0.000**
Moist Polar	**0.020**	**−0.002**	**0.019**	n/a	n/a	n/a	**0.015**
Moist Tropical	0.000	0.001	0.691	n/a	n/a	n/a	0.931
Transition	−0.009	0.001	0.484	n/a	n/a	n/a	0.153

Air mass analysis could not be run without smoothing (n/a = not applicable). Results with p≤0.05 are shown in bold. Mean values for events and non-events are air temperature (T) and dew point temperature (T_d_) departures from the long-term daily mean in z-score units. Air mass values are mean frequencies based on a 7-day centered moving average filter. The z-score values for events in the 1-sample test are the same as in column 2.

Because the high number of non-event days *vs.* event days can bias the *t*-test, an additional test was performed comparing the 5-day smoothed weather variables during events to the long-term mean. Here, 1200 UTC temperature and dew point were both significantly lower during high P&I mortality events (Table 3).

For the daily SSC analysis, lower frequencies of moist moderate (MM) air (p = 0.019) and higher frequencies of moist polar (MP) air (p<0.001) occurred 15–19 days before high mortality events (Table 3). No relationship was found for dry polar (DP) air (p = 0.277).

### Episodic Analyses

**Figure 3:**
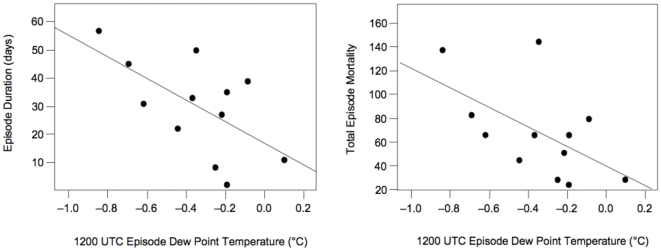
Scatter plots of pneumonia and influenza mortality episode duration and total mortality vs. dew point temperature. a) (left) Total episode duration (days) *vs.* mean episode dew point temperature (°C) (r = −0.61). b) (right) Total episode mortality (in z-score units) *vs.* mean episode dew point temperature (°C) (r = −0.56). The regression line shown in both graphs is for the least squares linear regression of the full data set. Both of these relationships were determined to have statistically significant slopes based upon 10,000 bootstrapped samples.

Temperature, dew point temperature, and air mass frequencies were examined 17 days prior to and throughout each of the 12 high P&I mortality episodes identified from 1975–2002. There is a statistically significant negative relationship between episode duration and mean 1200 UTC dew point (r = −0.61, p<0.05; Figure 3a). The longest episode (57 days in 1981) was associated with a dry period during which the mean dew point was more than 0.8°C below normal for that time of year. Total episode mortality is likewise negatively correlated with 1200 UTC dew point (r = −0.56, p<0.05; Figure 3b). Two of the three episodes with the lowest mortality also had dew points that were near normal, whereas the higher mortality episodes exhibited drier conditions. No significant relationships were found for afternoon variables, air temperature, or any of the SSC air mass types.

## Discussion

Dew point temperature is commonly used by atmospheric scientists to measure humidity because it is relatively invariant to pressure and temperature changes and thus is a conservative quantity. In New York City from 1975–2002, periods with high P&I mortality were preceded 2–3 weeks by periods with low morning dew points. Furthermore, for the 12 high mortality episodes identified in that period, morning dew point was negatively correlated with both episode duration (r = −0.61) and total episode mortality (r = −0.56). This finding of a linkage between dry air and influenza mortality is consistent with the results of recent research [6] showing that absolute humidity (a correlate of T_d_) influences the timing of the onset of the influenza season in various U.S. states (including New York state). Our results provide some limited evidence supporting laboratory studies linking dry air to higher airborne infection rates among guinea pigs [4].

The association between high frequencies of Moist Polar air masses prior to high mortality events is consistent with the dew point results. The average morning dew point in Moist Polar air in New York City is lower than for any air mass other than Dry Polar (Table 4). Although it may seem counter-intuitive that a “moist” air mass has a low dew point temperature, dew point must (physically) be less than or equal to air temperature, so cold air masses typically have low dew points, especially in winter. In general, Moist Polar air masses are relatively uncommon in the cold season in New York, occurring only 7.3% of the time (Table 4) and are associated with cold, overcast and often stormy conditions with moist air arriving from the Atlantic Ocean [33].

**Table 4:** SSC air mass frequencies, mean 4 a.m. dew point temperatures, and ranks for October–March for New York City (La Guardia).

AIR MASS	FREQUENCY	RANK	MORNING T_d_	RANK
**DP**	25.9	2	−9.3	1
**MP**	7.3	4	−2.0	2
**DM**	31.2	1	−1.5	3
**MM**	13.6	3	3.5	5
**DT**	1.7	6	−1	4
**MT**	6.9	5	9	6

We also identified a significant relationship between low Moist Moderate air mass frequencies 3–4 weeks before mortality episodes. In an effort to understand this association, we calculated the correlation between Moist Moderate frequencies and the other air mass types during that period. Moist Moderate is negatively correlated with the two driest air masses—Dry Polar (r = −0.47) and Moist Polar (r = −0.31) (Table 4). Thus, the significant association between low Moist Moderate frequencies prior to P&I mortality events appears to be a proxy for the cool, low dew point conditions that are common when Moist Polar and Dry Polar air masses are present. On average, cold season dew points are 5.5°C higher in Moist Moderate air masses than in Moist Polar (Table 4).

The lack of a direct Dry Polar relationship is surprising, as Dry Polar air masses exhibit the most extreme combination of cold air and low humidity. Dry Polar is far more common than Moist Polar in New York winters, however, so its high daily frequency during the influenza season limits the likelihood of identifying an underlying relationship. It might be more fruitful to examine an extreme cold, dry subset of Dry Polar air masses to identify the coldest and driest days.

### Conclusions

In New York City, high P&I mortality periods within a given year were preceded by multiple day periods with unusually low temperature and humidity. Over the 28-year period of this study, we identified 12 episodes of high P&I mortality and found that both the total mortality occurring during each episode and duration of each episode were inversely correlated with the average morning dew point temperature prior to and during the episodes. These results support the burgeoning hypothesis that unusually cold dry air enhances the airborne transmission of influenza virus.

The exploratory nature of this analysis was necessitated by the lack of an underlying theory of influenza seasonality, socio-behavioral factors, and inherent variability in disease transmission and virulence. The time between infection and a resulting mortality event (i.e. “latency”) varies between individuals depending on age, overall health, co-morbid conditions, and other factors. Thus, lags must be estimated to best fit the overall data structure. Similarly, the high frequency variability in the variables requires some smoothing to elucidate relationships, and the selection of appropriate smoothers is somewhat subjective. Nevertheless, our findings are consistent with several others. For example, there is evidence supporting a two-week lag between rising influenza virus and pneumonia mortality [28]. Other research showed fairly convincingly the existence of a 2–4 week lag between laboratory-confirmed cases of influenza and increased incidence of invasive pneumococcal disease [43]. The weather variables used in this study do not directly account for the ambient conditions experienced by the influenza victims while indoors, but cold and/or stormy weather could result in the decedents spending more time in heated indoor environs with low humidity, thereby enhancing infection opportunities [8].

For this study, P&I mortality was used to characterize the influenza time series in New York City. The limitation of this method is the potential for confounding as P&I mortality includes mortality from infections other than influenza. In addition, in non-pandemic years, P&I mortality is skewed by the extremes of age. This limitation is unlikely to be a major contributor in this study as 90% of influenza-related deaths involve persons over the age of 65 during seasonal epidemics [45] and our mortality data are age-adjusted to account for demographic changes in New York City over the period of the study.

We chose to focus on New York City because the large population (and thus large daily P&I mortality rate) enhances statistical robustness, and New York weather is highly variable owing to its midlatitude, coastal location. These results should be confirmed using a similar methodology in other cities worldwide to determine if the humidity-influenza linkage is pervasive. It would be particularly interesting to determine how these relationships evolve in subtropical or tropical climates where the P&I mortality seasonality is more muted or nonexistent.

It is likely that the underlying causes of influenza seasonality are multi-factorial, and we suspect that weather is but one of those factors. A predictive model for P&I mortality based on weather alone would likely be unsuccessful in accounting for most of the short-term influenza variability. Nevertheless, our results confirm recent emerging hypotheses of a relationship between cold, dry air and influenza transmission or virulence [6], [8]. Identifying periods of low dew point temperatures a few days or even weeks in advance is well within the skill of existing weather forecast models. Given the lag between infection and mortality, it seems reasonable to propose that, during the prime influenza season, skillful forecasts of high P&I mortality periods could be made weeks to months in advance.

## Supporting Information

Figure S1 **Sample of mortality data from December 1, 1998 through March 10, 1999.** Daily mortality (z-scores) (red dashed lines) shows evidence of the beginning of a prolonged peak starting around day 43. When these data are smoothed using a 17-day centered moving average filter (solid red line), the mortality peak becomes more evident. In our analysis, we instead employ a leading 17-day smoother (blue line), which effectively shifts the red line forward by 8 days. High mortality episodes are classified when the z-scores exceeds 1, so the 1999 episodes begins on day 39 and ends on day 77.
(TIF)

Figure S2 **Example of weather data treatment for 1998–1999.** (Bottom panel) Raw dew point temperature (blue dashed line), dew point z-score (green line) and z-scored after application of a 5-day centered moving average smoother (solid black line). (Top panel) Days classified as having a moist moderate air mass present (vertical bars), moist moderate frequency anomalies to remove seasonality (red dashed line), and smoothed using a 7-day centered moving average filter (purple line). For the air mass variable, this procedure converts a nominal variable into a continuous variable for subsequent analysis.
(TIF)

Figure S3 **Comparison of smoothed mortality (black dashed line) to smoothed dew point temperature (red line) for the 1998–1999 season.** The decline in dew point on day 10 preceded the start of the mortality increase by approximately 17 days. During the subsequent period of declining dew points, mortality continued to rise. When dew point reached its minimum for this period on day 31, 17-day lagged mortality was one standard deviation above the mean. Although there is no consistent 1∶1 lagged relationship between dew point temperature and mortality, this example illustrates the procedure and shows a general linkage between a low dew point period in mid-late December, 1998 and a subsequent high pneumonia and influenza mortality anomaly several weeks later.
(TIF)
