# Supplementary figures and images for: The Impact of Weather on Influenza and Pneumonia Mortality in New York City, 1975–2002: A Retrospective Study
Source: PLoS One. 2012 Mar 28;7(3):e34091. doi: 10.1371/journal.pone.0034091 (PMC3314701; doi:10.1371/journal.pone.0034091)

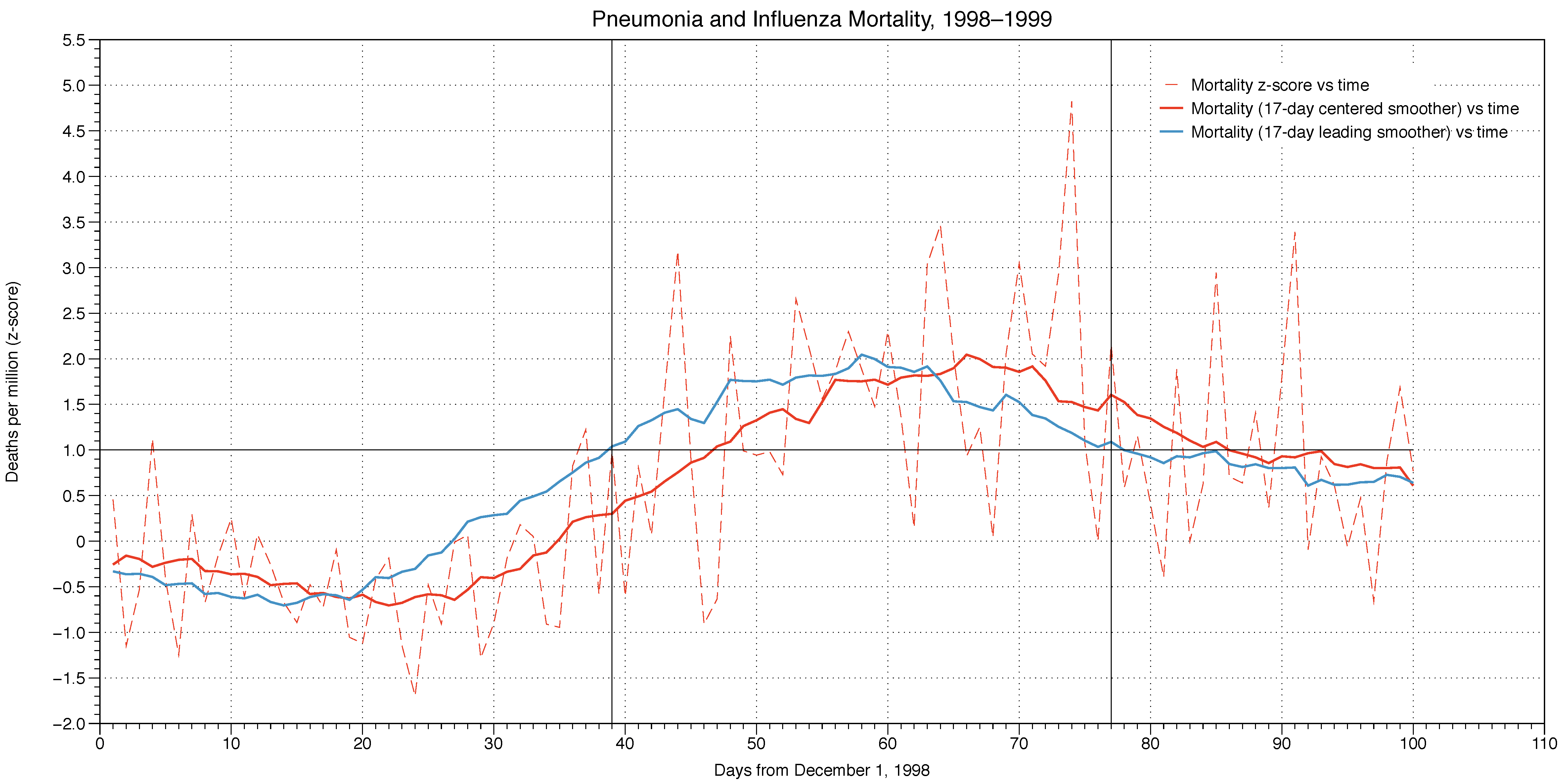

Supplement: Figure S1 — Sample of mortality data from December 1, 1998 through March 10, 1999. Daily mortality (z-scores) (red dashed lines) shows evidence of the beginning of a prolonged peak starting around day 43. When these data are smoothed using a 17-day centered moving average filter (solid red line), the mortality peak becomes more evident. In our analysis, we instead employ a leading 17-day smoother (blue line), which effectively shifts the red line forward by 8 days. High mortality episodes are classified when the z-scores exceeds 1, so the 1999 episodes begins on day 39 and ends on day 77. (TIF) [file pone.0034091.s001.tif]

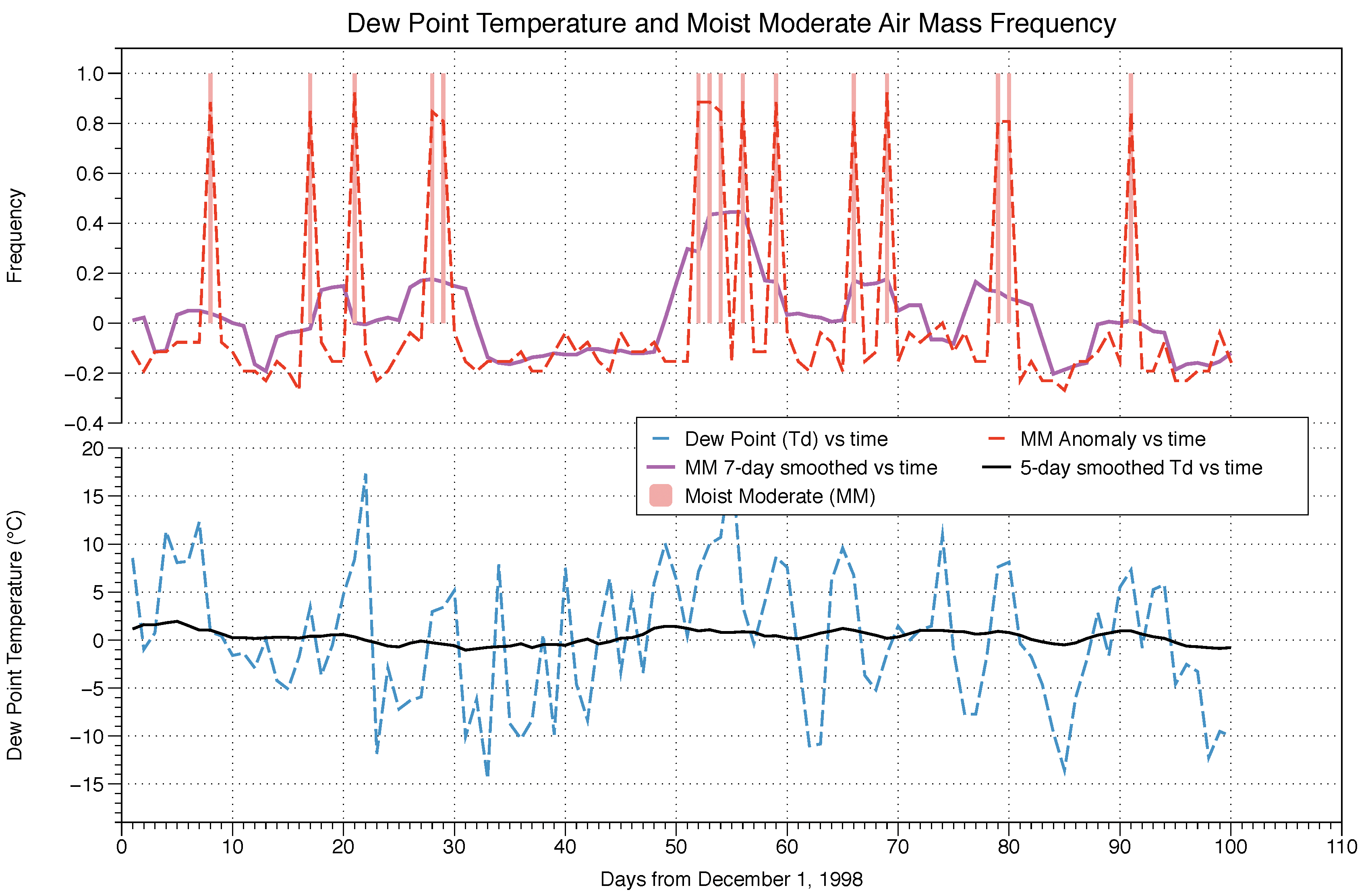

Supplement: Figure S2 — Example of weather data treatment for 1998–1999. (Bottom panel) Raw dew point temperature (blue dashed line), dew point z-score (green line) and z-scored after application of a 5-day centered moving average smoother (solid black line). (Top panel) Days classified as having a moist moderate air mass present (vertical bars), moist moderate frequency anomalies to remove seasonality (red dashed line), and smoothed using a 7-day centered moving average filter (purple line). For the air mass variable, this procedure converts a nominal variable into a continuous variable for subsequent analysis. (TIF) [file pone.0034091.s002.tif]

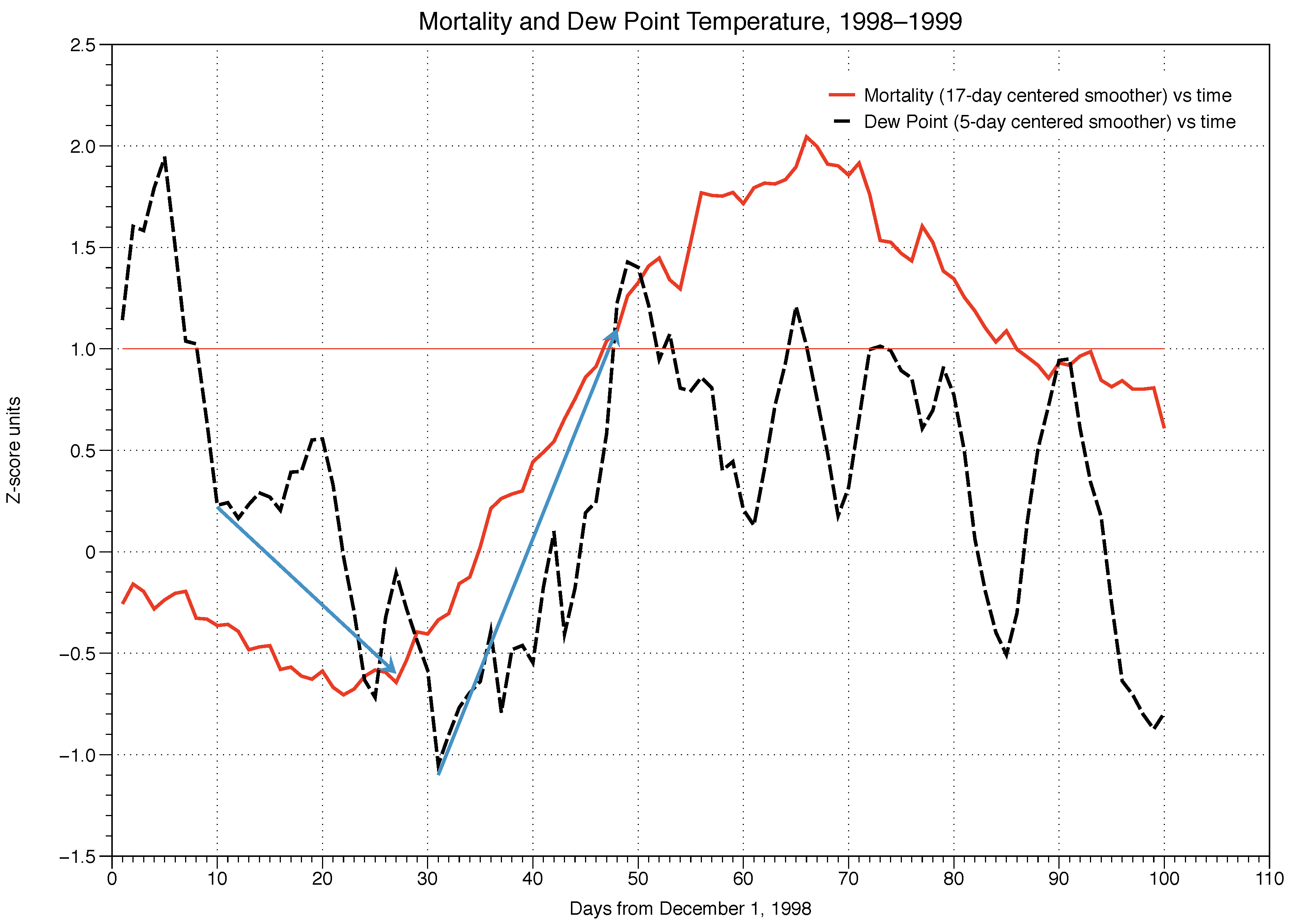

Supplement: Figure S3 — Comparison of smoothed mortality (black dashed line) to smoothed dew point temperature (red line) for the 1998–1999 season. The decline in dew point on day 10 preceded the start of the mortality increase by approximately 17 days. During the subsequent period of declining dew points, mortality continued to rise. When dew point reached its minimum for this period on day 31, 17-day lagged mortality was one standard deviation above the mean. Although there is no consistent 1∶1 lagged relationship between dew point temperature and mortality, this example illustrates the procedure and shows a general linkage between a low dew point period in mid-late December, 1998 and a subsequent high pneumonia and influenza mortality anomaly several weeks later. (TIF) [file pone.0034091.s003.tif]
